# Functional impact of allelic variations/haplotypes of TNF-α on reproductive tract infections in Indian women

**DOI:** 10.1038/s41598-020-79963-y

**Published:** 2021-01-12

**Authors:** Vineeta Sharma, Subash Chandra Sonkar, Pallavi Singhal, Anoop Kumar, Rakesh Kumar Singh, V. G. Ramachandran, Roopa Hariprasad, Daman Saluja, Mausumi Bharadwaj

**Affiliations:** 1 Division of Molecular Genetics and Biochemistry, National Institute of Cancer Prevention and Research (ICMR), I-7, Sector 39, Noida, Uttar Pradesh 20130 India; 2 Multidisciplinary Research Unit (MRU), Maulana Azad Medical College, University of Delhi, New Delhi, India; 3 Dr. B.R. Ambedkar Center for Biomedical Research (ACBR), University of Delhi (North Campus), New Delhi, India; 4 Division of Clinical Oncology, National Institute of Cancer Prevention and Research (ICMR), Noida, Uttar Pradesh India; 5 Department of Microbiology, University College of Medical Science, Delhi University, Delhi, India; 6 National Institute of Biologicals, A-32, Secror 62, Noida, Uttar Pradesh India; 7 Directorate of Census Operations, Goa, India

**Keywords:** Biochemistry, Cancer, Microbiology, Molecular biology, Diseases

## Abstract

The aim of the present study is to investigate the functional role of TNF-α single-nucleotide polymorphisms/haplotypes in an association with reproductive tract infections (RTIs) in symptomatic and asymptomatic women. A total of 850 consecutive subjects consisting of 400 cases and 450 healthy controls, were screened for RTIs, along with their risk factors and associated symptoms. The propensity score matching was performed to reduce the confounding bias arise owing to covariates and to balance the data between two groups. A total of 211 pairs (1:1) have been created. Genotyping of rs1800629 (-308) and rs361525 (-238) SNPs of TNF-α was done by PCR–RFLP followed by sequencing. The functional implication of TNF-α SNPs in an association with RTIs was also checked by using ELISA. The frequency of -238A allele and -308A allele was found to be twofold *(P* < *0.0001*) and threefold *(P* < *0.0001)* higher in the presence of RTIs. AA haplotype emerged as a major player in an association with RTIs and elevated TNF-α expression. The present study revealed the functional role of rs1800629 (-308) and rs361525 (-238) of TNF-α in an association with RTIs. This information may be used to establish biomarkers for an inflammatory response during the persistence of RTIs.

## Introduction

Reproductive tract infection (RTIs), infection of the reproductive genital tract, are second most common cause for loss of healthy life in sexually active and reproductive age in developing countries^1^. The burden of untreated RTIs is high in women because of these infections are often asymptomatic or the symptoms are not recognizable. The morbidity and mortality related to RTIs divest society of important contributions made by women in terms of economic, social, and cultural development. Although RTIs affect women in both developing and industrialized countries, the infections and their sequelae are women health problem in poor resource settings countries around the world. The most commonly prevalent RTIs due to sexually transmitted diseases (STDs) like *Chlamydia trachomatis* (CT), *Neisseria gonorrhoeae* (NG), *Trichomonas vaginalis* (TV), *Syphilis* and it have more significant in the past decade with the emergence of Human immunodeficiency virus (HIV) and Acquired immunodeficiency syndrome (AIDS)^2^. The Global Burden of various RTIs incidence and prevalence are varying between countries to countries and even between regions within a country. Disease and Injury estimated around 27.4% disability-adjusted life years were lost from *Chlamydia* and *Neisseria*. Globally, about 20% of women aged under 24 years have a prevalent Human Papillomavirus (HPV) infection^2^. The worldwide incidence of ovarian cancer in 2012 (239,000 cases), uterine cancer (320,000 cases) and cervical cancer (528,000 cases) which were caused by the (HPV) infection and categorized into the greatest of all life- menacing diseases^3^. Increased burden of RTIs due to most infections are asymptomatic and remain untreated RTIs. As per published epidemiological data, among pregnant women in developing countries, it is estimated that gonorrhea rates are 10–15 times higher, *Chlamydia* rates are 2–3 times higher, and *Syphilis* rates are 10–100 times higher than the rates of STDs among women in industrialized countries^4^**.** The female reproductive tract has the cell type (epithelium) that capable for the host resistance to pathogen by serving as a physical barrier to invading pathogen or by producing certain cytokines^5,6^. Some STDs cause damage to the epithelium. For example, in CT infection, epithelial cells become infected and are targeted by adaptive and innate immune responses. The infections on these cells are control of cell-mediated immunity which is regulated by cytokines that are secreted from T- helper cells.

Cytokines are a group of immune proteins which are implicated in immunity, inflammation and also protect against infections. TNF-α is a proinflammatory cytokine, it plays an important role in many inflammatory diseases^7^. It is located on chromosome 6p21.231 in the polymorphic region of MHC-III. The TNF-α expression is generally harmonized at the transcriptional level and during cell cycle regulation^8^. Various studies showed the association of TNF-α with many human diseases like cervical cancer^9,10^, renal cell carcinoma^11^.

TNF-α being a potent pro-inflammatory cytokine, considered in control of infections. Several single-nucleotide polymorphisms (SNPs) have been identified primarily in the promoter region of TNF. TNF α polymorphism was focused in many studies on rs1800629 (-308) and rs361525 (-238), which have been widely studied as an impending determinant of disease susceptibility. In several studies, the TNF-α has been associated with various cancers, such as Oral cancer^12^, breast carcinoma^13^, most important cervical cancer^9,14^ and renal cell carcinoma^11^.

However, with the superlative comprehension, no report is available to address the role of TNF-α promoter polymorphism in the development of RTIs such as *CT, NG, TV* and HPV worldwide. Therefore, the present study was designed to investigate the impact of TNF-α genotypes/haplotypes on RTIs prevalence. We also analyzed the association of TNF-α genotypes/haplotypes with TNF-α expression as well as with various demographic characteristics.

## Materials and methods

### Sample collection

In the present study the association of SNPs at -308 (G/A) and -238 (G/A) of TNF-α promoter with the susceptibility to RTIs was evaluated in a total of 850 consecutive subjects consisting of 400 symptomatic women from STIs/RTIs clinic and 450 healthy control subjects (women undergo routine check up in Gynecology clinic). After performed propensity score matching number of cases and controls for the study were 422 (in which 211 cases and 211 controls). From collaborated hospitals, Guru Teg Bahadur Hospital, Delhi and District Hospital, Noida. The age and ethnicity-matched controls having no history of any disease and with normal cervical cytology were included as a control in the present study. The women were then subjected to per speculum examination, cervical scrapes collected from endocervical canal using cytobrush for the detection of RTIs by the molecular method. The blood sample was also collected at the same time from cases and controls. A questionnaire which was filled at the time of sample collection to study their socio- demographic, sexual and reproductive history, lifestyle, abortion, contraception and any symptoms of genital infections details. A written informed consent was taken from all the subjects (cases and controls) before enrollment in the study. The study was approved by the ethical committee of the institution (National Institute of Cancer Prevention and Research—NICPR) and was carried out in accordance with the principles of Helsinki declaration (World Medical Association, 2000).

### DNA extraction and PCR detection of multiple RTIs

High molecular-weight genomic DNA was extracted from fresh cervical scrape samples by the standard method with proteinase K digestion followed by phenol/chloroform/isopropanol treatment^15^. The quality and quantity of DNA was estimated by using NanoDrop ND-1000 spectrophotometer (Thermo Fisher Scientific, Waltham, MA, USA) and extracted DNA was stored at −20 °C for further use. Quality of genomic DNA of all samples was checked by β-globin gene amplification by PCR, which was used as internal control.

Then extracted DNA was used in detection of RTIs including HPV, *CT, TV* and NG by polymerase chain reaction (PCR) using type specific primers^9,14^. HPV diagnosis was performed by using consensus primers MY09 and MY11^16^, PCR for testing the presence of *CT, NG* and *TV* using specific PCR based assay as described as per the published protocols^11,14–16^.

### SNP genotyping of TNF-α Genes by PCR–RFLP

We used polymerase chain reaction-restriction fragment length polymorphism (PCR–RFLP) approach to genotype these two loci of the TNF-α promoter region with modification as employed by Jang et al.^17^. The two amplicons (-308G/A and -238G/A) were digested overnight at 37 ^ͦ^ C by NcoI and BglI respectively, according to the method mentioned in a previous study from our laboratory^9^. The digested products were run on 10% native polyacrylamide gels.

### DNA sequencing

20% of samples were randomly sequenced to validate the data generated by PCR–RFLP method. According to the protocol of conventional dideoxy chain termination method using ABI Prism 310 automated DNA Squencer (Applied Biosystem, USA) sequencing reactions were performed. Similar results were observed by both the techniques.

### Enzyme-linked immunosorbent assay (ELISA) for TNF-α

TNF-α concentration in human serum were measured by enzyme-linked immunosorbent assay (ELISA) using Human TNF-α ELISA Kit according to manufacturer protocol (BOSTER-Immunoleader (Cat. No. EK0525)] (https://www.bosterbio.com/human-tnf-alpha-picokine-trade-elisa-kit-ek0525-boster.html). Kit have a TNF-α coated plate; standard, standard diluents, control, biotinylated anti-TNF-α antibody, PBS buffer, ABC working solution, TMB substrate and TMB stop solution. Briefly, 100 μl of human serum samples along with diluted standard/controls were added in TNF-α coated wells and incubate for 2 h followed by addition of 100 μl biotinylated anti-TNF-α at room temperature. Excess unbound analyte and secondary antibody was removed by the washing of plate three times. ABC working solution conjugate was used to detect bound antibody by adding a chromogen substrate TMB solution which result into the development of blue color complex. TMB stop solution was used to stop color reaction which results into development of yellow color product and absorbance recorded at 450 nm. The TNF-α concentration in samples standard and controls was evaluated on the basis of intensity of color complex (Table 1).

**Table 1:** Characteristics of studied population in symptomatic and asymptomatic women (before and after propensity score matching).

Covariate	Before PS matching					After PS matching (balanced covariates)		
	Cases (%) N = 400	Controls (%) N = 450	χ^2^ P-value	OR	(95% CI) P-value	Cases (%) N = 211	Controls (%) N = 211	χ^2^ (P-value)
Age in years (SD)	34.9 (9.2)	30.7 (10.7)	< 0.001^a^	–	–	32.7 (8.6)	32.5 (10.7)	0.82^a^
**Age (years) in groups**								
Less than 30	126 (31.5)	209 (46.4)	< 0.001	0.534	(0.400–0.702) < 0.0001	89 (42.2)	80 (37.9)	0.37
30 and above	274 (68.5)	241 (53.6)		Ref		122 (57.8)	131 (62.1)	
**Marital status**								
Married	387 (96.8)	439 (97.6)	0.48	1.341	(0.593–3.028) 0.6169	207 (98.1)	206 (97.6)	0.74
Divorced/widow	13 (3.2)	11 (2.4)		Ref		4 (1.9)	5 (2.4)	
**Abortion**								
Yes	290 (72.5)	143 (31.8)	< 0.001	5.660	(4.210–7.609) < 0.0001	110 (52.1)	114 (54.0)	0.70
No	110 (27.5)	307 (68.2)		Ref		101 (47.9)	97 (46.0)	
**Contraceptive status**								
Yes	194 (48.5)	97 (21.6)	< 0.001	3.615	(2.675–4.884) < 0.0001	72 (34.1)	72 (34.1)	1.00
No	206 (51.5)	353 (78.4)		Ref		139 (65.9)	139 (65.9)	
**Tobacco status**								
Yes	381 (18.5)	69 (15.33)	0.218	5.660	(4.210–7.609) 0.2543	31 (14.7)	37 (17.5)	0.43
No	326 (81.5)	381 (84.67)		Ref		180 (85.3)	174 (82.5)	
**Education status**								
Education	113 (28.2)	188 (41.8)	< 0.001	3.539	(2.656–4.717) < 0.0001	72 (34.1)	64 (30.3)	0.40
Un-education	287 (71.8)	262 (58.2)		Ref		139 (65.9)	147 (69.7)	
**Socio economic status**								
Low	74 (59.8)	178 (39.6)	< 0.001	2.268	(1.723–2.987) < 0.0001	102 (48.3)	108 (51.2)	0.56
Middle	161 (40.2)	272 (60.4)		Ref		109 (51.7)	103 (48.8)	

χ^2^ refers p-value fromChi-square test; a based on Anova; Ref. represents reference category; OR refers unadjusted odds ratio; 95% CI represents 95 percent confidence interval. After PS matching represents the outcomes after subgroup analysis ofpropensity score matching (balanced covariate).

### Statistical analysis

By using statistical software PLINK v.1.07 (http://pngu.mgh.harvard.edu/purcell/plink) the data analysis was performed and to compare the distributions of TNF-α polymorphism between symptomatic and healthy controls, GraphPad InStat version 3.0. Chi-square test/Fisher’s exact test (for smaller numbers on subgroup analysis) was used. The risk estimates were calculated for dominant, codominant and recessive genetic models by using the most common homozygous genotype as reference. The odds ratio (OR) and its 95% confidence intervals (CIs) was also calculated as a measure of the association between these three polymorphisms markers by using PLINKv.1. 07 (http://pngu.mgh.harvard.edu/purcell/plink). Linkage Disequilibrium (LD) estimates were determined by the significance of statistical chi-square/Fisher’s exact test was considered as two-tailed. *P* value < 0.05 was considered as significant. Genotypes were further checked for the conformance of Hardy Weinberg equilibrium. The statistical power of the study determined by using Quanto software (http://hydra.usc.edu/gxe/) was > 80%. The logistic regression analysis was also performed to investigate the role of adjusted odds ratio with controlled of demographic and socio-economic variables in the study (Table 2).

**Table 2:** Prevalence of different Reproductive Infections in cases and controls.

Reproductive	Before propensity score matching					After PS matching (balanced covariates)	
	Cases N = 400 (%)	Controls N = 450 (%)	OR (95% CI)	AOR (95% CI)	P-value	OR (95% CI)	P-value
Infections							
**Human Papillomavirus (HPV)**							
Positive	59 (15)	13 (3)	5.816 (3.138–10.779)	6.487 (3.152–13.351)	< 0.001	5.22 (2.116–12.900)	< 0.001
Negative	341 (85)	437 (97)	Ref	Ref		Ref	
**Chlamydia Trachomatis (CT)**							
Positive	73 (18)	17 (4)	5.686 (3.290–9.826)	4.507 (2.402–8.455)	< 0.001	4.772 (2.240–10.165)	< 0.001
Negative	327 (82)	433 (96)	Ref	Ref		Ref	
**Neisseria Gonorrhea (NG)**							
Positive	21 (5)	7 (2)	3.506 (1.474–8.339)	3.235 (1.178–8.886)	< 0.001	2.484 (0.859–7.180)	0.093
Negative	379 (95)	443 (98)	Ref	Ref		Ref	
**Trichomonasvaginalis (TV)**							
Positive	43 (11)	12 (3)	4.396 (2.283–8.463)	4.109 (1.924–8.776)	< 0.001	4.772 (2.240–10.165)	< 0.001
Negative	357 (89)	438 (97)	Ref	Ref		Ref	

OR (95% CI) means odds ratio with 95% confidence interval, AOR refers adjusted odds ratio for age, socio-economic status education status, contraceptive status, abortion, tobacco status and marital status along with 95% confidence interval by using logistic regression analysis.

### Propensity score matching

Propensity score matching analyses was performed in STATA (version 13) software (STATA Corporation, College Station, TX, USA). The propensity score approach was initially introduced by the Rosenbaum and Rubin (1983)^17^, it helps to minimize the possible bias arising from the study design, therefore, we constructed the PS model to adjust for the possible confounding variables for having an outcome or not^18,19^. The covariates used for the calculation of the propensity score were age, marital status, abortion, tobacco status, contraceptive status, education status and socioeconomic status (Table 1). Cases and controls were individually matched at a ratio of 1:1 by using the nearest matching method within a caliper distance. Caliper distance selects matching control whose propensity score is nearer to case subject with the proper limitation that the absolute difference in the propensity score must not be below the pre-fixed threshold^20,21^. Accordingly, the unmatched samples have been removed from the study whose propensity score could not match due to the greater caliper distance^21^. Total, 211 cases and 211 controls have been generated by using propensity score matching.

## Results

### Population characteristics

The Demographic characteristics of the studied population are listed in Table 1. The mean age of the symptomatic and controls were 34.9 ± 9.2 and 30.7 ± 10.7 years, whereas after propensity score matching, mean age of cases is 32.5 years and 32.7 years for controls. In symptomatic women, 86% of women were got married below the age of 21 years while in controls the frequency was only 26%. We also noticed that the percentage of women having higher parity and history of abortions were also significantly higher in cases. Besides, we also noticed that the women who delivered child under age of 21 years the age more prone to have RTIs. We also noticed that illiteracy, use of contraceptives, lower socio- economic status and menstrual irregularities are some of other factors which significantly put their role in RTIs.

### Prevalence of different RTIs in symptomatic and asymptomatic women

The Frequency of reproductive tract infections like HPV, *CT, NG* and *TV*, in the studied population has been presented in Table 2. We have found 49% (196/400) were positive for multiple RTI infections in symptomatic women, but in healthy controls, the prevalence of different RTIs was 12% (49/450).

The prevalence of HPV was found to be significantly (*P* < 0.0001) higher in symptomatic women (15%) as compared to controls (3%). After PS matching (with balanced covariates) the predicted odds ratio of the HPV was found more likely (OR = 5.22, 95% CI 2.116–12.900) statistically significant in cases as compared to controls. *Chlamydia* infection was revealed 18% (73/400) in cases but in controls, it was found to be 4% (17/450) (*P* < 0.0001). The predictable OR of infection was found to be at 5.6 fold (OR = 5.686, 95% CI 3.290–9.826; *P* = 0.0001) AOR = 4.560, 95% CI 2.427–8.565) higher in cases as compared to controls. *Neisseria*, was prevalent in 5% (21/400) cases, while 2% (7/450) in controls. The frequency of *Trichomonas* was found 11% (43/400) in cases, rather 3% (12/450) in controls. Observations showed that all four RTIs were statistically significant in cases as compared to controls in before and after propensity score matching.

### Association of clinical symptoms with RTIs

**Figure 1:**
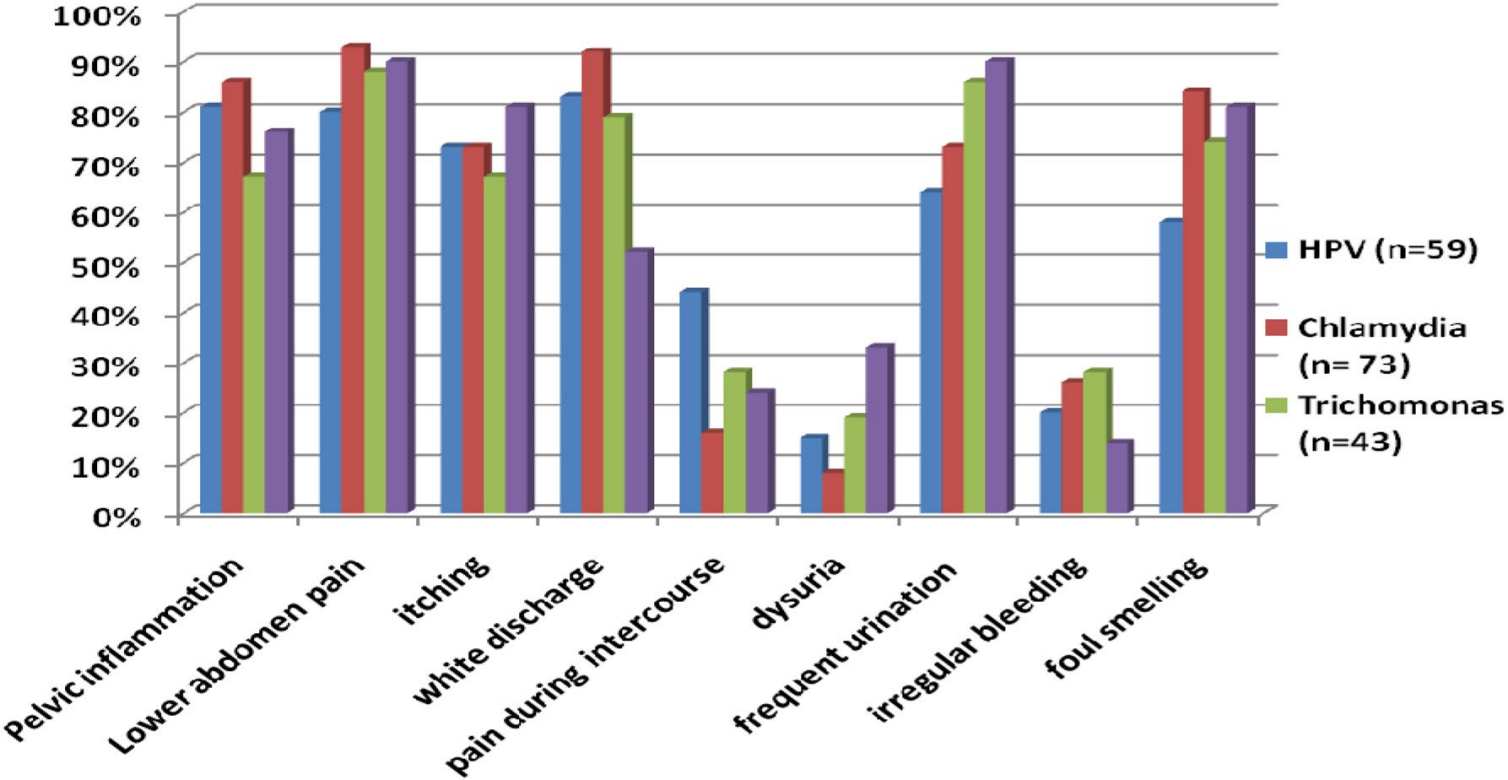
Association of different symptoms in patients infected with HPV and *Chlamydia trachomatis, Trichomonas vaginalis* and *Neisseria Gonorrhea* in cases.

We also tried to find out the association of clinical symptoms with infections Fig. 1. We observed that > 60% of the RTIs (HPV/*CT/TV/NG*) cases, pelvic inflammation, lower abdominal pain, and itching was noticed. Whereas white discharge was found to be more associated with (*CT/TV*/HPV). The women complaining frequent urination have the highest probability to be infected with *NG* than after *TV* and *CT. CT/NG* infected women are more likely to be having foul smelling discharge. On the other hand, we could not find any significant association of pain during intercourse, dysuria and irregular bleeding with any of RTIs.

### Genotypic analysis of SNPs in the TNF-α Locus

The genotypic distribution and allelic frequencies of TNF**-**α promoter SNPs rs1800629 (-308) and rs361525 (-238) in the cases and control population are depicted in Table 3. We used three different types of genetic models (dominant, recessive, and codominant) to evaluate the association of TNF**-**α genotype with RTIs. The Genotypic frequencies of both polymorphisms were checked and found to be in Hardy–Weinberg equilibrium in cases and controls (*P* < 0.05).

**Table 3:** Distribution of TNF- α rs1800629 (-308) & rs361525 (-238) genotypic/Allelic frequency between cases and controls.

Samples	-238 (rs361525)			-308(rs 1,800,629)		
	Controls (N = 450)	Cases (N = 400)	P-value	Controls (N = 450)	Cases (N = 400)	P-value
**Genotyping**						
GG (%)	279 (60%)	133(33%)	–	286(64%)	74(19%)	–
GA (%)	115 (25%)	123(31%)	–	87(19%)	209(52%)	–
AA (%)	66 (15%)	144(36%)	–	77(17%)	117(29%)	–
**Odds ratio (95% CI)**						
**Dominant Model (GG vs GA + AA)**						
Before PS match	Reference	2.984(2.253–3.950)	< 0.0001	Reference	7.682(5.594–10.549)	< 0.0001
After PS match*	Reference	3.268(2.179–4.902)	< 0.0001	Reference	8.291(5.203–13.214)	< 0.0001
**Recessive Model (GG + GA vs AA)**						
Before PS match	Reference	0.305(0.219–0.425)	< 0.0001	Reference	0.499(0.360–0.692)	< 0.0001
After PS match	Reference	0.265(0.1662–0.4223)	< 0.0001	Reference	0.701(0.450–1.092)	< 0.0001
**Codominant Model (GGvs.GA)**						
Before PS match	Reference	0.462(0.332–0.641)	< 0.0001	Reference	0.107(0.075–0.154)	< 0.0001
After PS match	Reference	0.455(0.285–0.726)	< 0.0001	Reference	0.083(0.048–0.141)	< 0.0001
**Codominant Model (GGvs.GA)**						
Before PS match	Reference	0.226(0.158–0.324)	< 0.0001	Reference	0.170(0.115–0.250)	< 0.0001
After PS match	Reference	0.1882(0.112–0.314)	< 0.0001	Reference	0.194(0.112–0.336)	< 0.0001
**Allelic Association**						
G	653	389		659	357	
A	247	411		241	443	
Odds ratio (95% CI)	Reference	2.793(2.283–3.417)	< 0.0001	Reference	3.393(2.769–4.156)	< 0.0001

After PS match*represents odds ratio after performing propensity score matching with balanced covariates (covariates included were age, marital status, socio-economic status, education status, abortion, contraceptive status, tobboco status).

### Genotypic distribution of TNF-α -238G/A polymorphism (rs361525) and risk of RTIs

The genotypic distribution of GG, GA and AA genotypes at -238 loci were 60%, 25%, and 15% in controls while in cases it was 33%, 31% and 36% respectively (Table 3).. When we compared to control group with the cases, the frequency of the carrier genotype (GA + AA) was observed significantly higher (*P* < 0.0001, OR = 2.984, 95% CI 2.25–3.95) in cases when a dominant model was taken into consideration. After PS matching (with balanced covariates) the odds ratio of the carrier genotype (GA + AA) was found more likely (OR = 3.26, 95% CI 2.179–4.902) statistically significant in cases as compared to controls The frequency of AA genotype was found more than twofold higher in cases (36%) as that of in control group (15%). We also noticed the frequency of A allele significantly higher in cases *(P* < *0.0001).* These findings illustrated the positive effect of A allele in the development of RTIs.

### Genotypic distribution of TNF-α -308 G/A polymorphism (rs1800629) and risk of RTIs

The frequency of GG, GA, AA genotype of TNF-α -308 G/A locus were 64%, 19% and 17% in controls while 19%, 52% and 29% of cases, respectively. The difference in the frequency of carrier genotype (GA + AA) between cases and controls was found statistically significant in the dominant model. When we compared the genotypic distribution between control group and cases significant p- values was observed, it was interesting to note that there is increment of an frequency of variant AA genotype from control group (17%) to cases (29%), Differences in the distribution of allelic frequencies of G and A allele was observed in cases, the differences were found significant. We found the association of RTIs and -308 G/A polymorphism.

### Haplotypes and progression of RTIs

Haplotype analysis using statistical software Haploview and PLINK showed the presence of 4 haplotypes which has been shown in Table 4. All the haplotypes were presented in > 1% of the population studied. On analysis, it was observed that the frequency of GG haplotype was significantly higher in controls which show the possible protective role of GG haplotype in RTIs. On the other hand, the frequency of AA, GA, and AG haplotypes were found significantly higher in cases. In which, AA haplotype was revealed as highly associated with the risk of RTIs.

**Table 4:** Distribution frequencies of TNF-α (rs361525) & (rs1800629) haplotypes between cases and control.

S. no.	Haplotypes (-238 G/A & -308 G/A) N = 850, Frequency	Cases N = 400	Controls N = 450	*P* value	OR
1	**GG : 379; 0.446**	100 (25)	279 (62)	3.2 × 10^–33^	2.31
2	**AA: 199; 0.234**	127 (32)	72 (16)	2.2 × 10^–14^	2.35
3	**GA: 142; 0.167**	94 (24)	48 (11)	7.1 × 10^–13^	1.95
4	**AG: 129; 0.152**	79 (19)	51 (11)	1.68 × 10^–6^	0.295

The associations of minor allele SNPs in haplotypes with cases were found to be interesting. The minor (rs361525) -238A allele was found in two haplotypes (AA and **A**G) out of 4. Both haplotypes showed significant values for cases (*P* < *0.001*, OR 2.31). The minor (rs1800629) -308A alleles were also found in two haplotypes (AA and G**A).**

### Haplotypic distribution and RTIs

Furthermore, we were also interested to find out the association of AA haplotypes with different RTIs studied in the present study. Interestingly, we found that the frequency of AA haplotype was the highest among three of the risk haplotypes (AA, GA, AG) in HPV infected women. Table 5. We also noticed same for the rest of other infections such as *Chlamydia, Trichomonas,* and *Neisseria*. Above results again expose AA haplotype as one of the major risk factors in an association with RTIs.

**Table 5:** Distribution frequencies of TNF-α (rs361525) and (rs1800629) haplotypes among different RTIs.

Haplotypes	HPV		*Chlamydia*		*Trichomonas*		*Neisseria*	
	Positive (N = 72)	Negative (N = 778)	Positive (N = 90)	Negative (N = 760)	Positive	Negative	Positive	Negative
GG (379)	11 (3%)	368 (97%)	11 (3%)	368 (97%)	8 (2%)	371 (98%)	2 (1%)	377 (99%)
	**P < 0.0001**		**P < 0.0001**		**P < 0.0001**		**P < 0.0001**	
AA (199)	50 (25%)	149 (75%)	50 (25%)	149 (75%)	33 (17%)	166 (83%)	19 (10%)	182 (90%)
	**P < 0.0001**		**P < 0.0001**		**P < 0.0001**		**P < 0.0001**	
GA (142)	7 (5%)	135 (95%)	9 (6%)	133 (94%)	7 (5%)	135 (95%)	3 (2%)	139 (98%)
	P = 0.82		P = 0.8219		P = 0.3842		P = 0.1366	
AG (129)	4 (4%)	126 (96%)	16 (12%)	114 (88%)	5 (4%)	125 (96%)	3 (2%)	127 (98%)
	**P = 0.0039**		**P = 0.0039**		P = 0.1234		P = 0.4021	

### Serum concentration of TNF-α

**Figure 2:**
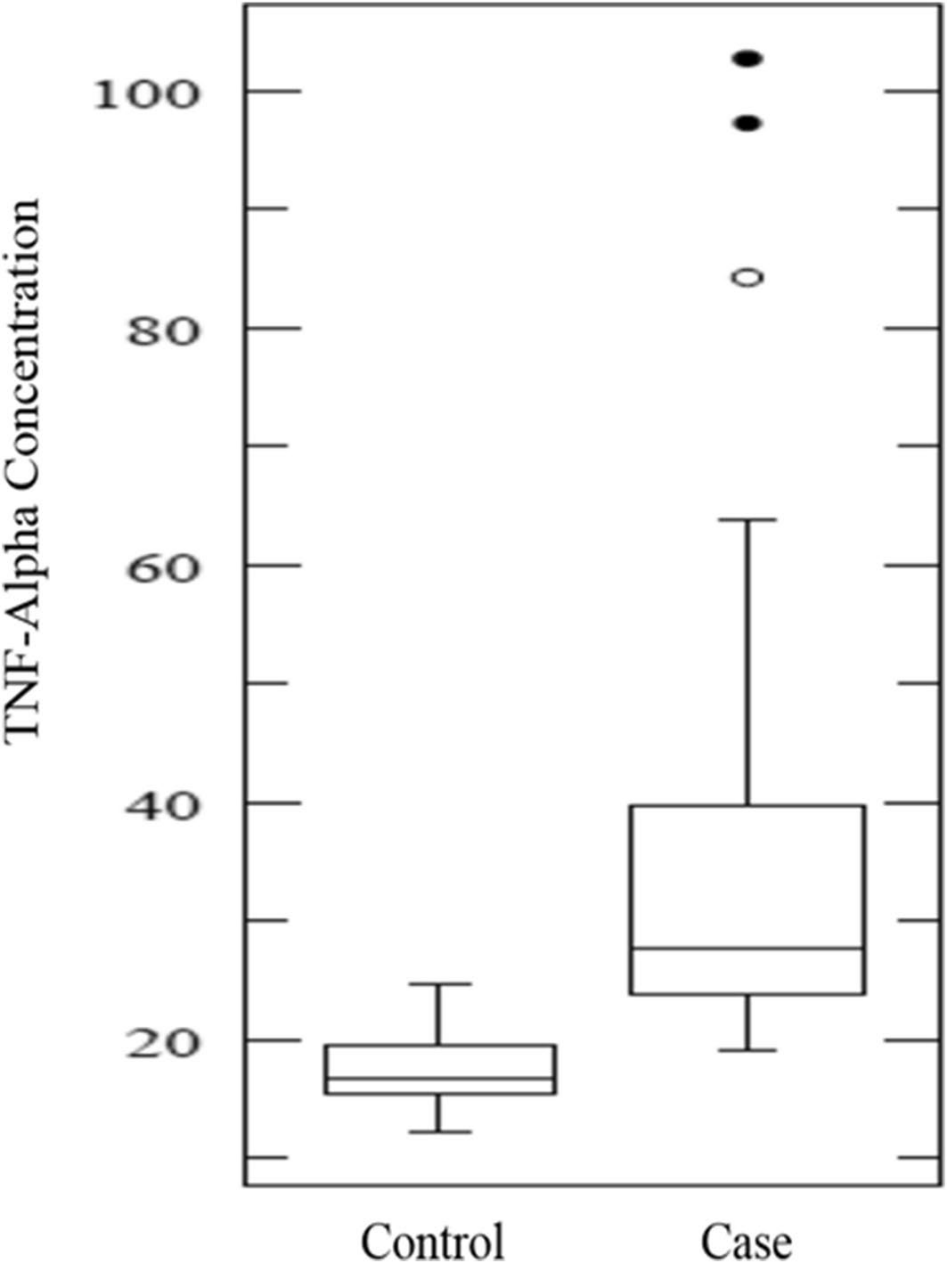
TNF-α concentration in cases and controls.

**Figure 3:**
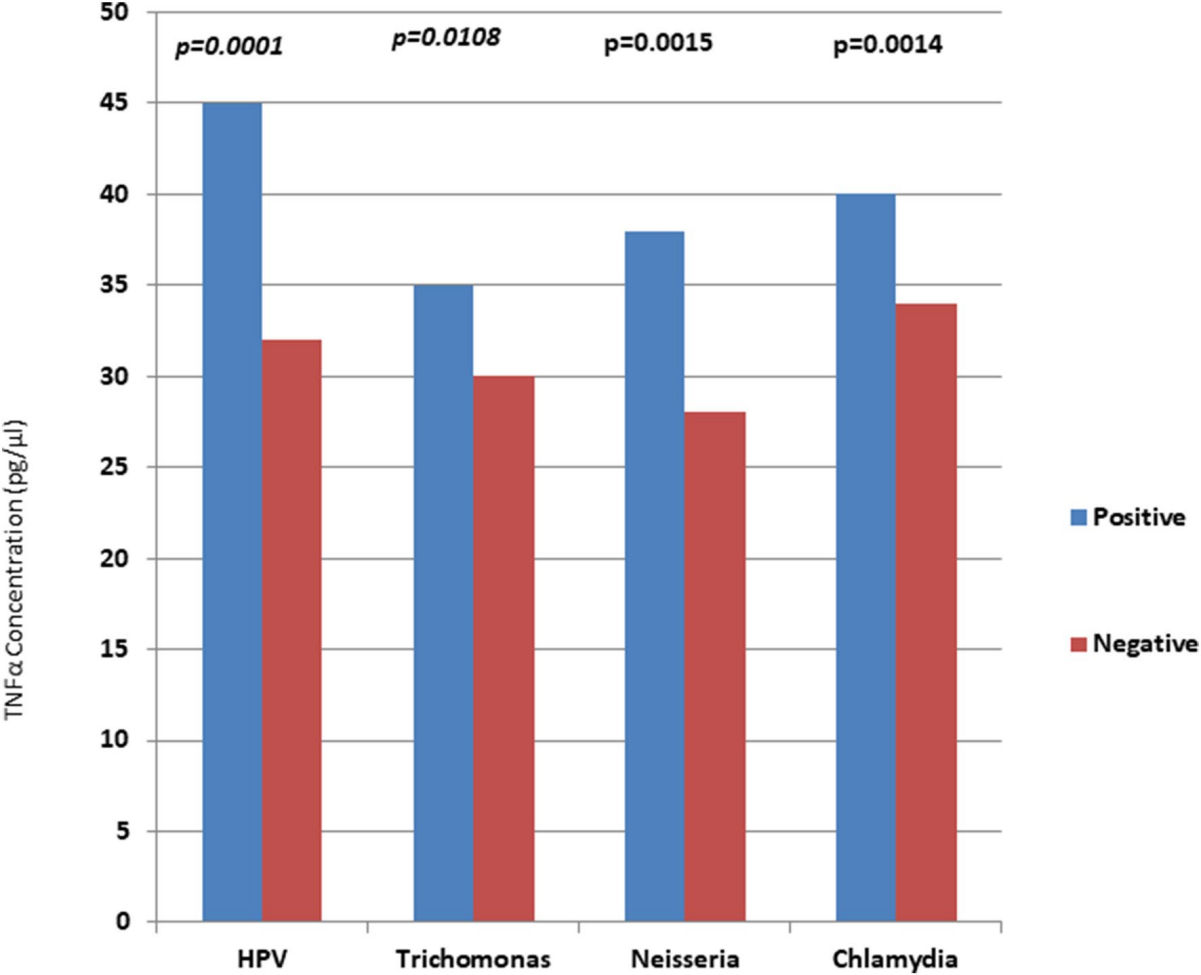
Bar diagram showing the prevalence of RTIs with TNF-α concentration.

The total serum concentration of TNF-α has been presented in Fig. 2. We noticed a significant increase in serum concentration of TNF**-**α from controls to cases by using ANOVA test (p < 0.0001). Furthermore, we also checked the TNF**-**α concentration with RTIs. Figure 3. Interestingly, we found the significantly higher concentration of TNF**-**α in presence of RTIs. i.e. *CT/TV/NG/*HPV.

**Figure 4:**
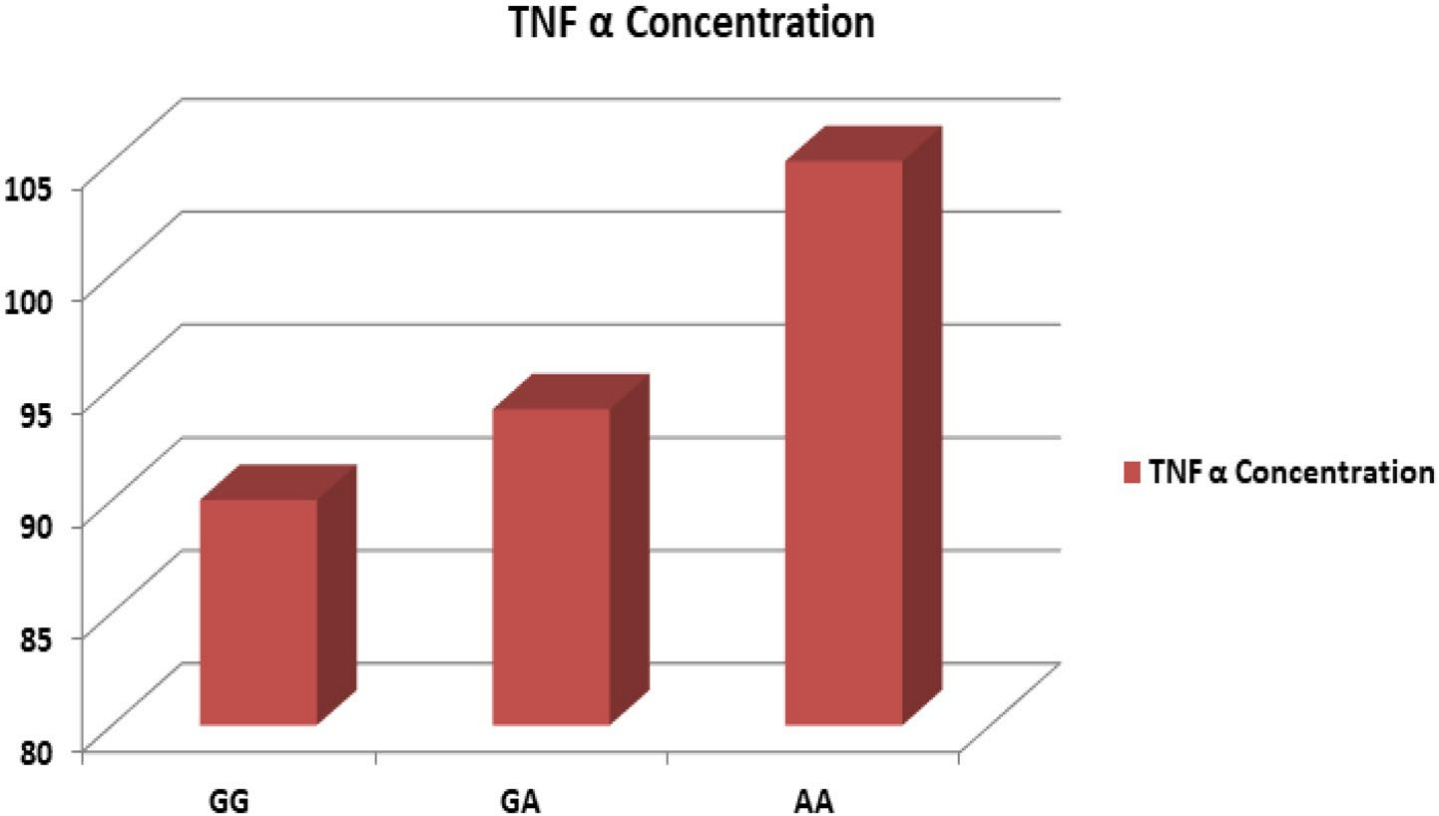
Bar diagram showing TNF-α concentration in association of GG, GA and AA haplotype.

We were also interested to know the impact of risk haplotypes with TNF-α concentration Fig. 4. After analysis, we observed that the concentration of TNF-α was the highest in presence of AA haplotype. These findings illustrate the positive impact of TNF-α polymorphisms on the elevated level of TNF-α expression.

## Discussion

Reproductive tract infections (RTIs) affect sexual and reproductive health worldwide. It is ranked one of the top five disease categories for which adults seek health care. According to the estimates of World Health Organization (2016) more than one million people acquire RTIs every day that result in an estimated 357 million new cases of STIs every year worldwide. Around 10–30% women cannot clear the infections because of low immunity, thus establishing the critical role played by other factors including host-genetic, immunological and viral factors associated with reproductive infections^22^.

This genetic susceptibility can be ascribed to the single nucleotide polymorphisms (SNPs) in the immunomodulatory TNF locus. TNF polymorphisms are found to be associated with huge range of inflammatory and immunomodulatory diseases^23^ because of inconsistency in the host immunogenic conditions in the form of sporadic polymorphisms in the immunomodulatory genes possibly will be a significant determinant for conferring cell mediated immunity (CMI) to reproductive tract infections.

Therefore, in the current study, we have evaluated the prevalence of RTIs and their correlation with SNPs in TNF-α rs1800629 (-308) and rs361525 (-238) locus among Indian women. We also observed their functional implications in association with *Chlamydia, Trichomonas*, *Human Papillomavirus* and *Neisseria* infections.

In the present study, the prevalence of *Chlamydia trachomatis (CT), Trichomonas vaginalis (TV)*, *Neisseria gonorrhea (NG)* and Human Papillomavirus (HPV) infections were found statistically significantly higher (*P* < *0.0001*) in symptomatic women. The frequency of *chlamydia* and HPV infection was found ~ sixfold higher in cases while for *TV* and *NG* it was ~ fourfold higher. The prevalence of *Chlamydia* infection was found 18% in cases which is in good agreement with two previous studies from Iran and Europe. They also showed that frequency of *CT* were 16% and 13.4% in Iranian^24^ and European populations^25^ respectively. Besides, there is also another study which found higher prevalence of *CT* in symptomatic women i.e. 30% which is higher than our study^26^. But in contrast, several other studies found lower frequencies of *CT* i.e. 7.5 and 9% in Scottish and Brazilian populations respectively^27,28^.

In this study the prevalence of *TV* was noticed 11% in cases. This finding is in concordance with several previous studies^29,30^ from different regions of the world, while in contrast to another study in Syrian population^31,32^.

Regarding prevalence of HPV, we found 15% in symptomatic women which is in good agreement with the study from European population^33^. The prevalence of HPV has been reported from region to region as it was shown lesser than 3% in Australia, New Zealand and United States while ~ 20% in India, Sub Saharan regions^34^ and in Chinese population^35^. A possible justification for these discrepancies may be attributable to geographic and climatic variations in different regions of world. Some of the studies documented a very low prevalence of *NG* in cases of European population^36^ while other studies reported a very high prevalence in American population^37^.

In the present study, we also tried to evaluate some risk factors in association with RTIs. On analysis, we noticed early age of marriage, higher parity, and history of abortions, illiteracy, menstrual irregularities, lower socio-economic status and the use of oral contraceptives as some of the significant *(P* < *0.0001)* risk factor associated with *CT, TV, NG* and HPV infection which is similar to our previous study in HIV infected women^38^. Besides, several other studies are in good agreement with our findings^39^.

Furthermore, significant association was also observed for clinical symptoms with reproductive infections. More than 60% of women complaining such as pelvic inflammation, lower abdominal pain, itching and frequent urination were found at higher risk of RTIs. These findings are similar to our previous study^40^.

On analysis of polymorphisms data of TNF-α loci, it was revealed that TNF-α rs1800629 (-308) and rs361525 (-238) SNPs are significantly associated with the risk of RTIs. In present study, TNF-α -238 AA genotype was found 2.7 fold higher in cases (36%) as compared to controls. The finding is in good agreement with previous findings in osteoarticular tuberculosis from Chinese population^41^, Hepatocellular carcinoma in South Korean population and renal disease patients in Indian population^42^ while in contrast with a report on European population (UK)^43^. These discrepancies may be due to difference in experimental designing and region wise changes in environmental factors.

We also noticed the frequency of A allele significantly higher *(P* < *0.0001)* in cases. These findings illustrated that A allele may be associated with an increased risk with respect to reproductive tract infections. Women who were A allele carriers, presented a twofold increased risk of reproductive infections, in Hispanic population (US)^23^, Chinese population^44^ and North Indian population^45^.

Additionally, TNF-α -308A alleles were specifically found to be related to studied infections and it can be labeled as major susceptibility allele for the development of reproductive tract infections (*CT, TV*, HPV and *NG*) with 3.3 fold increased risk among Indian women. The significant association of -308G/A with infection in our study is in concordance with the previous study by our group in cervical, breast and oral cancer^9,12–14^. This finding is in contrast to a report from Europe (UK)^46^.

On genotypic analysis, we found AA genotype in -308 of TNF-α to be related to the increased risk of RTIs. It was observed 29% in cases while 17% in controls. These findings are in good concurrence of the previous finding in Tunisians population^47^.

To complement further, on the role of SNPs in TNF-α loci, rs1800629 and rs361525 two- locus haplotype were constructed and their distribution was compared with respect to disease severity as well as reproductive infections (*CT, TV*, HPV and *NG*) status. Haplotype AA was found to be at 2.3 fold increased risk of reproductive infections *(P* < *0.0001)* while GG haplotype was appeared as protective haplotype.

It was interesting to analyze the association of AA haplotype with the RTIs (*CT*, *TV*, HPV and *NG*). The analysis illustrates that the frequency of AA haplotype was more in presence of any of the reproductive tract infections. The significant differences were observed in haplotypes of infected patients in cases with respect to controls. Our study is in concordance with the studies from Tunisian^48^, Portuguese^49^ and Indian population^9^. But in Iranian population, it varies^50^.

The epithelial cells of the cervix have been implicated in mucosal immune responses. TNF has been reported to exhibit cytostatic and cytotoxic effects on certain tumors. The levels of the cytokines in circulation could affect the disease susceptibility and increase the risk of reproductive tract infections. To extend our interest, we also observed the serum concentration of TNF-α in association with infections. On analysis, we could find a positive association of serum concentration and RTIs which is alike with several previous studies^9^. Furthermore, we also noticed a significant increase in TNF-α concentration of subjects having AA haplotype. It has been shown that elevated level in serum may modulate the various immune responses towards the RTIs susceptibility. Thus, it can be concluded that increased TNF-α level exhibits an immunosuppressive effect which leads towards progression in RTIs.

Different results are achieved in different populations with different genetic backgrounds. The study may help in future to understand the role of ethnic and geographical factors of disease pathogenesis as variable results are observed in the different population.

As far as our knowledge is concerned, in Indian women, this is the first study showing the role of TNF-α -308 and -238 genotypes/ haplotypes with the susceptibility, development, and progression of multiple reproductive tract infections.

## Conclusion

The present study revealed the potential role of genetic variations in immunomodulatory TNF-α -308 G/A and -238 G/A SNPs, associated with reproductive tract infections in Indian population. So, it is suggested that TNFα rs1800629 (-308) and rs361525 (-238) polymorphism could serve as an important biomarker in Indian women for their susceptibility to RTIs as it may play a key role in alteration of TNF-α production and the inflammatory response during the course of the disease. Haplotype analysis revealed AA, GA and AG haplotypes as important ‘risk/susceptibility haplotype’ and GG as ‘protective haplotype’ for *CT, TV, NG* and HPV risk. Basically, reproductive infections can be cured by organizing screening programs on a regular basis. Genotype-level associated a functional correlation between TNF-α rs1800629 (-308) and rs361525 (-238) polymorphism and elevated plasma TNF-α levels in the reproductive infected women or in cases. These findings could be useful as a prediagnostic marker, improving treatment efficacy and effectiveness, which ultimately contribute to the discovery of personalized treatment.

## References

[1] Vishwanath S (2000). Syndromic management of vaginal discharge among women in a reproductive health clinic in India. Sex Transm. Infect..

[2] Smith JS, Robinson NJ (2002). Age-specific prevalence of infection with herpes simplex virus types 2 and 1: a global review. J. Infect. Dis..

[3] WHO. World Health Organization. Prevalance and Incidence of Selected Sexually Transmitted Infections. 2011.

[4] Griffin RG, Wilkinson TH, Hoff GL (1999). HIV surveillance: a dynamic, not static, process to assure accurate local data. Sex Transm. Dis..

[5] Doerflinger SY, Throop AL, Herbst-Kralovetz MM (2014). Bacteria in the vaginal microbiome alter the innate immune response and barrier properties of the human vaginal epithelia in a species-specific manner. J. Infect. Dis..

[6] Ferreira VH, Kafka JK, Kaushic C (2014). Influence of common mucosal co-factors on HIV infection in the female genital tract. Am. J. Reprod. Immunol..

[7] Bequet-Romero M, Lopez-Ocejo O (2000). Angiogenesis modulators expression in culture cell lines positives for HPV-16 oncoproteins. Biochem. Biophys. Res. Commun..

[8] Raabe T, Bukrinsky M, Currie RA (1998). Relative contribution of transcription and translation to the induction of tumor necrosis factor-alpha by lipopolysaccharide. J. Biol. Chem..

[9] Kohaar I (2007). TNFalpha-308G/A polymorphism as a risk factor for HPV associated cervical cancer in Indian population. Cell Oncol..

[10] Kohaar, I. *et al.* Impact of Haplotype TNF-LTA locus with Susceptibility to Cervical Cancer in Indian Population. *Obstet. Gynecol. Int. J. *(2014).

[11] Nakajima K (2001). Tumor necrosis factor-alpha gene mutations and genotype changes in renal cell carcinoma. J. Urol..

[12] Bandil K (2016). Impacts of TNF-LTA SNPs/haplotypes and lifestyle factors on oral carcinoma in an Indian Population. Mol. Diagn. Ther..

[13] Kohaar I (2009). Association of single nucleotide polymorphisms (SNPs) in TNF-LTA locus with breast cancer risk in Indian population. Breast Cancer Res. Treat..

[14] Kohaar, I. *et al.* Impact of Haplotype TNF-LTA Locus with susceptibility to cervical cancer in Indian Population. *Obst. Gynecol. Int. J.* (2014).

[15] Sambrook, R. A. *Molecular Cloninig: A laboratory manual; Cold Spring Harbor Laboratory Press 3, 2001*. (2001).

[16] Manos MM (1989). Use of polymerase chain reaction amplification for the detection of genital human papillomaviruses. Cancer Cells.

[17] PAUL R.ROSENBAUM, D. B. R. The central role of the propensity score in observational studies for causal effects. *Biometrika* **70**, 41–55 (1993).

[18] Williamson E, Morley R, Lucas A, Carpenter J (2012). Propensity scores: from naive enthusiasm to intuitive understanding. Stat. Methods Med. Res..

[19] Williamson EJ, Forbes A (2014). Introduction to propensity scores. Respirology.

[20] Austin PC (2009). Some methods of propensity-score matching had superior performance to others: results of an empirical investigation and Monte Carlo simulations. Biom. J..

[21] Xia Y (2018). A dietary pattern rich in animal organ, seafood and processed meat products is associated with newly diagnosed hyperuricaemia in Chinese adults: a propensity score-matched case-control study. Br. J. Nutr..

[22] Wang, S. S. & Hildesheim, A. Chapter 5: Viral and host factors in human papillomavirus persistence and progression. *J. Natl. Cancer Inst. Monogr* 35–40 (2003). 10.1093/oxfordjournals.jncimonographs.a003480 12807943

[23] Deshpande A (2005). TNF-alpha promoter polymorphisms and susceptibility to human papillomavirus 16-associated cervical cancer. J. Infect. Dis..

[24] Molaei, B. *et al.* The frequency of gonorrheal and chlamydial infections in Zanjanian women in 2013–2014. *Int. J. Reprod. Biomed. (Yazd. )* **15**, 75–82 (2017). PMC5405219 28462398

[25] Van Liere GAFS, Dukers-Muijrers NHTM, Levels L, Hoebe CJPA (2017). High proportion of anorectal chlamydia trachomatis and neisseria gonorrhoeae after routine universal urogenital and anorectal screening in women visiting the sexually transmitted infection clinic. Clin. Infect. Dis..

[26] Khattab RA, Abdelfattah MM (2016). Study of the prevalence and association of ocular chlamydial conjunctivitis in women with genital infection by Chlamydia trachomatis, Mycoplasma genitalium and Candida albicans attending outpatient clinic. Int. J. Ophthalmol..

[27] Costa-Lira, E. *et al.* Prevalence of human papillomavirus, Chlamydia trachomatis, and Trichomonas vaginalis infections in Amazonian women with normal and abnormal cytology. *Genet. Mol. Res.* **16**, (2017). 10.4238/gmr16029626 28453175

[28] Shone J (2016). A Scottish multi-centre service evaluation examining the prevalence and diagnosis of Trichomonas vaginalis in symptomatic women attending sexual health clinics. Int. J. STD AIDS.

[29] Bochner AF (2017). A cross-sectional analysis of Trichomonas vaginalis infection among heterosexual HIV-1 serodiscordant African couples.

[30] Mocumbi S, Gafos M, Munguambe K, Goodall R, McCormack S (2017). High HIV prevalence and incidence among women in Southern Mozambique: Evidence from the MDP microbicide feasibility study. PLoS ONE.

[31] Bristow, C. C. *et al.* Chlamydia trachomatis, Neisseria gonorrhoeae, and Trichomonas vaginalis screening and treatment of pregnant women in Port-au-Prince, Haiti. *Int. J. STD AIDS* 956462416689755 (2017). 10.1177/0956462416689755 PMC5837282 28134005

[32] Yentur DN (2016). Investigation of the prevalence of Trichomonas vaginalis among female Syrian refugees with the complaints of vaginitis aged between 15–49 years. Mikrobiyol. Bul..

[33] Chatzistamatiou K (2017). Diagnostic accuracy of high-risk HPV DNA genotyping for primary cervical cancer screening and triage of HPV-positive women, compared to cytology: preliminary results of the PIPAVIR study. Arch. Gynecol. Obstet..

[34] De, M. C., Plummer, M., Vignat, J. & Franceschi, S. Worldwide burden of cancer attributable to HPV by site, country and HPV type. *Int. J. Cancer* **141**, 664–670 (2017). 10.1002/ijc.30716 PMC5520228 28369882

[35] Wu, X. *et al.* [Prevalence of type-specific human papillomavirus infection among 18–45 year-old women from the general population in Liuzhou, Guangxi Zhuang Autonomous Region: a cross-sectional study]. *Zhonghua Liu Xing. Bing. Xue. Za Zhi.* **38**, 467–471 (2017). 10.3760/cma.j.issn.0254-6450.2017.04.011 28468064

[36] Toby, M. *et al.* Prevalence of porA pseudogene deletion among Neisseria gonorrhoeae isolates referred to the UK. *Sex Health* (2017). 10.1071/SH16162 28514990

[37] Park J (2017). Prevalence of Chlamydia trachomatis and Neisseria gonorrhoeae in an urban public hospital pregnancy termination clinic. Int. J. STD AIDS.

[38] Vineeta S (2015). Impact of oral contraceptives and smoking on the susceptibility of reproductive tract infections (RTIS) in immunosuppressed women: A hospital based. Int. J. Curr. Microbiol. App. Sci.

[39] Gaydos C, Hardick J (2014). Point of care diagnostics for sexually transmitted infections: perspectives and advances. Expert. Rev. Anti. Infect. Ther..

[40] Sonkar SC (2017). Evaluating the utility of syndromic case management for three sexually transmitted infections in women visiting hospitals in Delhi India. Sci Rep..

[41] Lv, Y. J. *et al.* Association of tumor necrosis factor-alpha gene polymorphism with osteoarticular tuberculosis prognosis in a Hebei population. *Genet. Mol. Res.* **15**, (2016). 10.4238/gmr15048937 27813605

[42] Sharma R, Agrawal S, Saxena A, Sharma RK (2013). Association of IL-6, IL-10, and TNF-alpha gene polymorphism with malnutrition inflammation syndrome and survival among end stage renal disease patients. J. Interferon Cytokine Res..

[43] Dominguez-Perez RA (2017). Association of cytokines polymorphisms with chronic peridontitis and rheumatoid arthritis in a Mexican population. Acta Odontol. Scand..

[44] Liu L (2012). Association between TNF-Î± polymorphisms and cervical cancer risk: a meta-analysis. Mol. Biol. Rep..

[45] Singh PK (2015). Association of TNF-alpha (-238 and -308) promoter polymorphisms with susceptibility of oral squamous cell carcinoma in North Indian population. Cancer Biomark..

[46] Mekinian A (2011). Functional study of TNF-alpha promoter polymorphisms: literature review and meta-analysis. Eur. Cytokine Netw..

[47] Roszak A, Misztal M, Sowinska A, Jagodzinski PP (2015). TNF-alpha -308 G/A as a risk marker of cervical cancer progression in the Polish population. Mol. Diagn. Ther..

[48] Sghaier I (2015). The relationship between TNF alpha gene polymorphisms (-238/-308), TNF RII VNTR (p75) and outcomes of hepatitis B virus infection in Tunisian population. Gene.

[49] Duarte I (2005). G-308A TNF-alpha polymorphism is associated with an increased risk of invasive cervical cancer. Biochem. Biophys. Res. Commun..

[50] Maddah M (2016). Association of tumour necrosis factor-alpha G/A -238 and G/A -308 single nucleotide polymorphisms with juvenile idiopathic arthritis. Int. J. Immunogenet..

